# Definitive-intent uniform megavoltage fractioned radiotherapy protocol for presumed canine intracranial gliomas: retrospective analysis of survival and prognostic factors in 38 cases (2013–2019)

**DOI:** 10.1186/s12917-020-02614-x

**Published:** 2020-10-31

**Authors:** M. Debreuque, P. De Fornel, I. David, F. Delisle, M. N. Ducerveau, P. Devauchelle, J. L. Thibaud

**Affiliations:** 1 Service de Neurologie, MICEN VET, Créteil, France; 2 Université de Toulouse, ENVT, Toulouse, France; 3 Service d’Oncologie et Radiothérapie, MICEN VET, Créteil, France; 4 GenPhySE, INRAE, Université de Toulouse, INPT, ENVT, Castanet Tolosan, France; 5 Hôpital Européen de Paris La Roseraie, Aubervilliers, France

**Keywords:** Brain tumour, Glioma, MRI, IMRT, 3D-Conformal radiotherapy, Fractionated radiotherapy, Megavoltage, Outcome, Prognosis, Quality of life

## Abstract

**Background:**

Radiotherapy (RT) is currently considered the treatment of choice for presumed canine intracranial gliomas. However, variable therapeutic responses are described, due to heterogeneous populations and different radiation methods or protocols. Only one study dedicated to intracranial suspected glioma highlighted prognostic criteria. Determination or confirmation of specific clinical and imaging prognostic factors may guide the therapeutic management of these tumours. The objectives were to provide data on long-term clinical outcome (including quality of life, QoL) and to determine specific prognostic factors associated with survival time. We report a single-institution retrospective study, including all dogs with suspected symptomatic primary solitary intracranial glioma, treated with a complete uniform fractionated megavoltage radiation protocol of 15x3Gy over 5 weeks, between January 2013 and February 2019. Thirty-eight client-owned dogs were included. Medical records were retrospectively evaluated for median overall survival time (MST), clinical and imaging responses. Prognostic factors on survival were researched in terms of signalment, clinical presentation, tumour imaging characteristics and response following RT. Finally, the RT’s impact on the dogs’ clinical signs and Qol were evaluated by the owners.

**Results:**

The disease-specific MST was 698 days (95% CI: 598–1135). Survival at 1 and 2 years were respectively 74.2 ± 7.4% and 49.0 ± 9.8%. Initial clinical signs were related to survival, as well as tumour characteristics such as cystic-pattern, mass effect and Tumour/Brain volume ratio. No significant adverse effect or radiotoxicity was observed.

**Conclusions:**

RT appears as a safe and effective treatment for canine intracranial gliomas, allowing long-term tumour control, improvement of life’s quality and management of associated clinical signs. The initial clinical signs and MRI characteristics (Tumour/Brain volume ratio, cyst-like lesion and mass effect) may help predict the prognosis.

## Background

Gliomas are the second most frequent primary brain neoplasm in dogs, accounting for approximatively 35% of all the central nervous system (CNS) primary tumours [1, 2]. They represent a pleomorphic group arising from glial cells and mainly includes astrocytomas and oligodendrogliomas [3, 4]. Most gliomas occur in adult dogs with median age of 8 years [1, 2]. Brachycephalic breeds such as Boxer, Boston terriers, French and English Bulldogs seem to be at risk [1, 2]. Histology is the gold standard for definitive diagnosis of tumour’s type and grade, but remains rare because of practical, financial and safety considerations. Routinely, the presumptive antemortem diagnosis of intracranial tumour is based on signalment data and compatible magnetic resonance imaging (MRI) (imaging modality of choice), or Computed Tomography (CT) characteristics.

Several treatments options exist: symptomatic treatment (glucocorticoids and anticonvulsants), cytotoxic chemotherapy, surgery, radiotherapy (RT) and more recently immunotherapy [5, 6] or even entotherapy [7] (image-guided intratumoural chemotherapy treatment). Currently, RT is considered the treatment of choice for intracranial tumours in dogs [8–11]. Its objective is to induce tumour cell death or inhibit further cell division, while minimizing damage to any normal tissue surrounding or in the irradiated volume. The majority of studies on irradiation of brain neoplasms includes all types of tumours, most cases being meningiomas [9, 12–20] and only one recent case study is exclusively dedicated to gliomas [10].

As reported in the largest studies on external fractionated megavoltage RT for animals with intracranial gliomas, the median overall survival time (MST) ranges from 226 to 430 days [9–11, 13] and disease-specific survival reaches 772 days [11]. These discrepancies may be due to heterogeneous populations of dogs and tumour types/grade, multiple radiation protocols and various evaluation criteria. According to recent publications, only initial clinical signs and relative tumour volume appear to be prognostic criteria [10, 13]. Hence, dogs with severe neurological signs (non-ambulatory state, dysphagia) [13] or depressed mentation [10, 13] have significantly lower survival time than dogs with no or mild clinical signs, and dogs with relative tumour volume under 5% of the total calvarial volume, live significantly longer [10]. However, these results are not systematically observed, and controversies persist on statistically significant criteria specific for gliomas [11, 14, 19, 20]. Furthermore, studies failed to demonstrate the prognostic significance of tumoural MRI features (pre-radiation MRI characteristics or tumour progression after RT) [14, 19, 20].

The aim of the present study was to provide data on long-term clinical outcome (including quality of life, QoL) in dogs with suspected primary solitary intracranial gliomas, solely treated with a uniformly fractionated megavoltage radiation protocol. The other objective was to determine glioma-specific prognostic factors (epidemiological, clinical and image-based) associated with survival time.

## Results

**Fig. 1:**
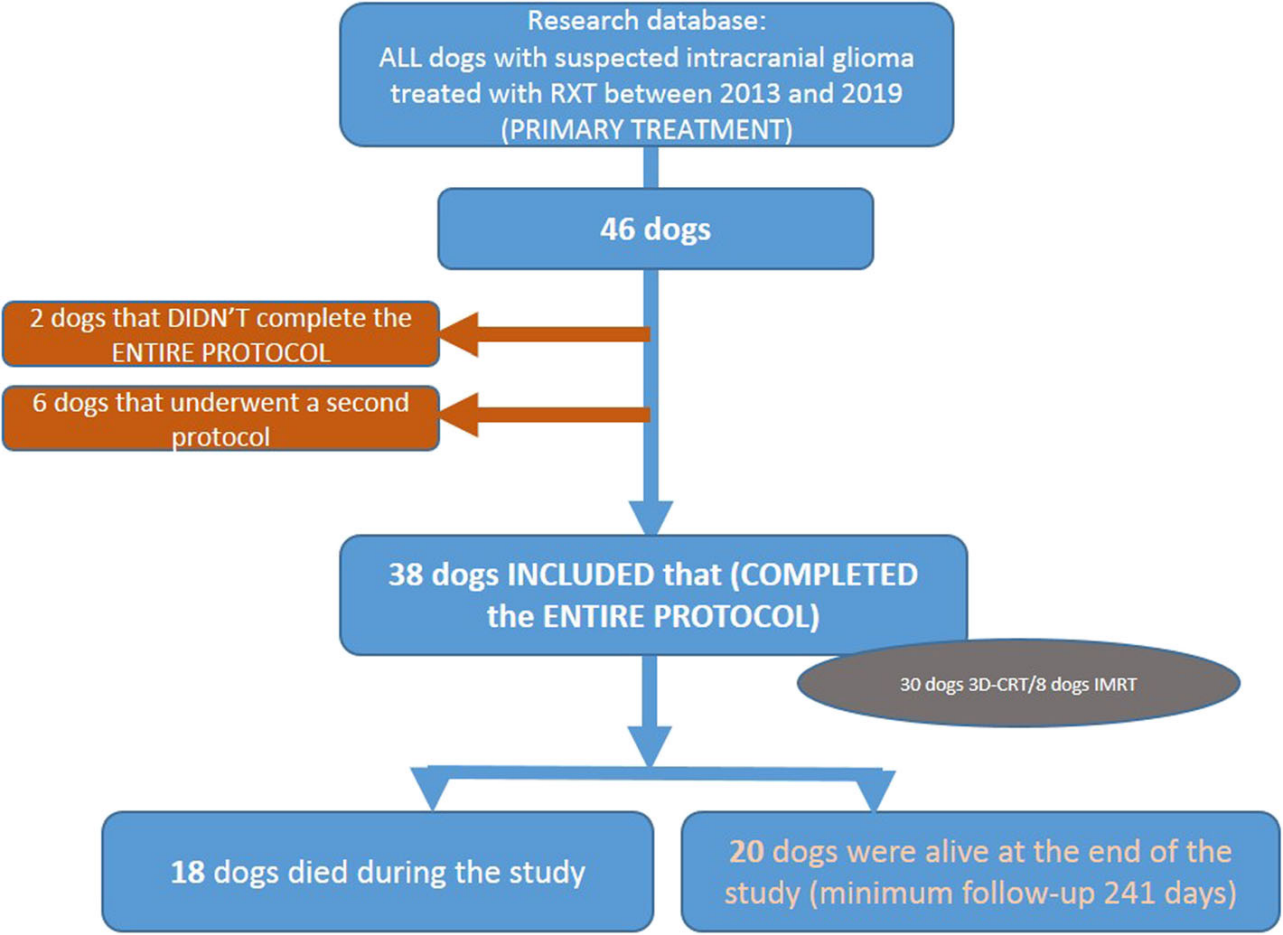
Diagram illustrating the study population in the per-protocol study design (Orange writings: censored animals)

Of the 46 dogs presented for radiation therapy for suspected symptomatic intracranial glioma, to the authors’ institution, 38 dogs met the inclusion criteria for the study. Two dogs were excluded because of treatment discontinuation due to neurological deterioration and MRI confirmation of tumour progression. Six dogs that underwent a second radiation protocol, because of local or metastatic recurrence, were also excluded (Fig. 1).

### Dogs and tumour characteristics

Thirty-eight symptomatic dogs were considered in the study: 14 intact males, 3 neutered males, 5 intact females and 16 neutered females, with a median age of 8.2 years (mean 8.1, range 4.5–12.5 years) and a median weight of 13.1 kg (mean 18.1, range 3.7–37 kg). All dogs were pure breeds. The cohort included 20 French bulldogs, 3 boxers, 3 English bulldogs, 2 Cane Corso dogs, 2 Maltese dogs and 1 each of 8 other breeds, which represented 27 brachycephalic dogs (71%), and 11 non-brachycephalic dogs (29%).

None of the dogs had evidence of life-compromising disease or unexpected metastases based on whole-body CT (Computed Tomography) scans. The main presenting complaints at the first clinical examination included seizures (35/38), behavioral changes (8/38), circling (6/38), altered mentation (5/38), proprioceptive deficits (3/38) and ataxia (2/38). Thirty-five dogs were presented with a history of seizures (92.1%) and 23 of them (23/35, 65.7%) showed seizures as their only sign of neurologic disease. Seizure frequency was detailed in 33 dogs, 16 dogs had clusters seizures and two had demonstrated status epilepticus (SE). During the first neurological examination, abnormalities were graded as absent in 23 dogs (seizures only), mild/moderate in seven dogs and severe in eight of 38 dogs. Neurologic signs were consistent with focal disease and the tumour location confirmed by MRI studies.

Thirty-seven tumours were supratentorial, and a single one was infratentorial. Specific localization was frontal for 15 cases, parietal/temporal for 20 cases, occipital for two cases and caudal fossa for one case (brain stem). The 37 dogs with supratentorial tumour had signs consistent with forebrain disease (circling, seizure, behavioral changes, altered mentation or proprioceptive deficits). The dog with infratentorial tumour showed ataxia and proprioceptive deficits. All MRI details are reported in Table 1.

**Table 1:**
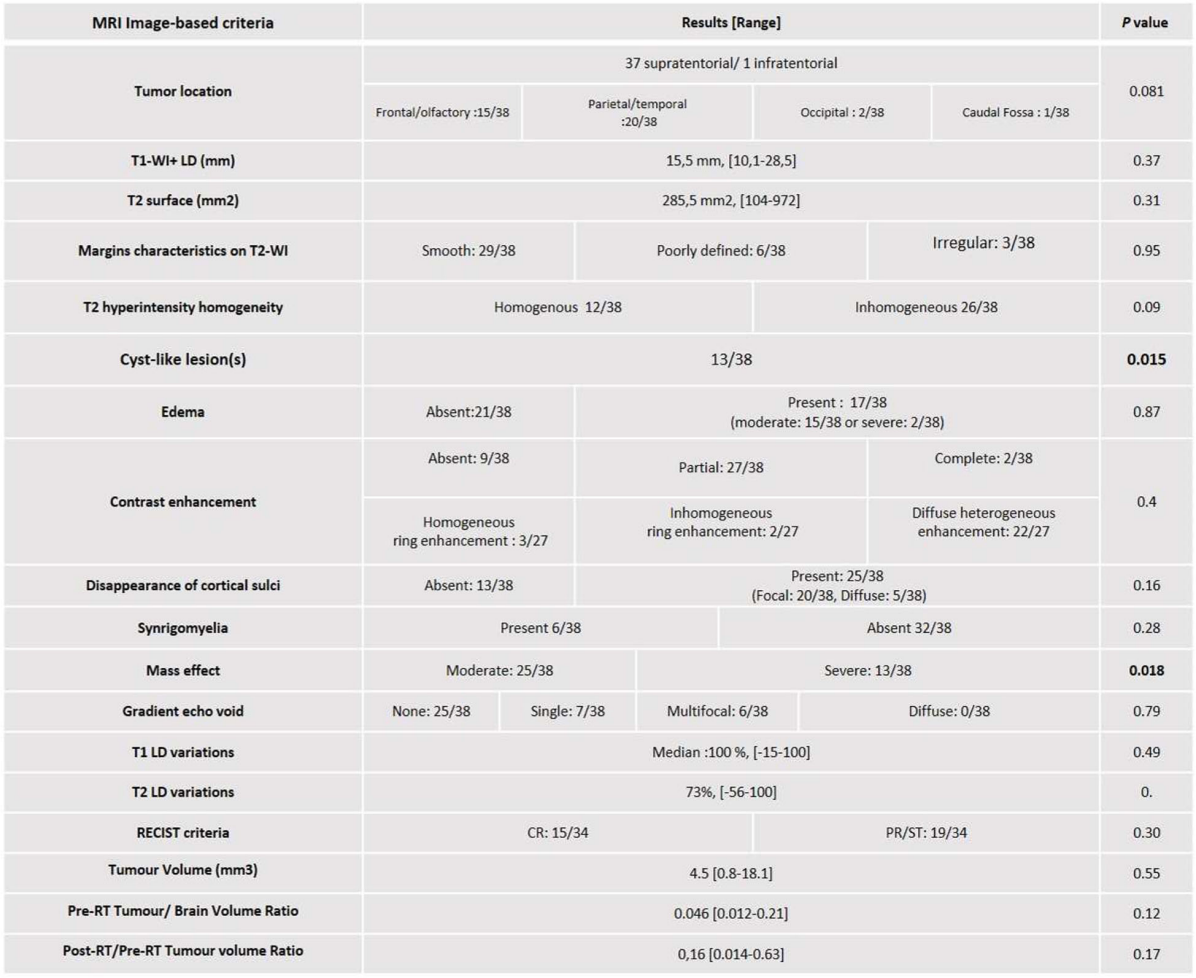
MRI image-based criteria used for statistical analysis. Results of univariable statistical analysis (KM and Log-rank tests for cross classified variables, Cox regression model and LRT test for continuous variables). MRI-cystic pattern, tumour location and mass effect have a significant effect on survival (*p* < .05)

### Treatment

Treatment with 15 × 3 Gy was planned for all dogs. The first radiation session occurred in a median of 10 days after the diagnostic MRI, and 5 days [Range 3–10] after the planning CT simulation. Thirty of the 38 dogs (78.9%) were treated with 3D-CRT (3-dimensional conformal radiation therapy) and 8/38 (21.1%) with IMRT (Intensity-modulated radiation therapy). A median of three fields was used for 3D-CRT (Range 3–5) and five for IMRT. No treatment interruption was reported.

All irradiated dogs received between 0.5 mg and 1 mg/kg/q24 of methylprednisolone, for between 1.5 and 6 months, gradually tapered after the first MRI-recheck. No direct life-threatening secondary steroid effects were noticed, even if some dogs showed mild polyuria-polydipsia, typical pot-belly appearance and diffuse truncal alopecia.

All the patients, which presented with seizures, received anticonvulsant treatment, either in monotherapy (24/35) or in a combination therapy (11/35): Phenobarbital (24/35), Imepitoin (5/35), Potassium bromide (4/35) or Levetiracetam (13/35). They were maintained during the entire duration of the follow-up period and adapted to the seizure frequency and anticonvulsants serum levels for Phenobarbital and Potassium Bromide.

All animals were followed for at least 49 days to a maximum of 1492 days, and at least 241 days until 1492 days for alive dogs at the end of study. The median duration of follow up was 561 days. At the end of the study, 18/38 dogs (47.3%) were dead, 20 dogs (52.7%) were alive (Fig. 1).

### Clinical follow-up and outcome

The majority of dogs having completed the RT, were re-evaluated (clinical and MRI control) for the first time, approximatively 3 months after the last session of irradiation, then every 2 to 6 months. Twenty dogs had at least a second follow-up MRI. Ten dogs had more than three follow-up MRIs.

A significant majority of dogs showed initial neurologic signs improvement within the 5 weeks of the RT (proportion of dogs with improved neurologic signs = 0.86 (95% CI: 0.76–0.97, *p* value < 0.001). In detail, 34/38 dogs showed improved neurologic signs after protocol completion, corresponding to 19 dogs with only seizures as initial signs, and 15 dogs with neurologic signs with/without seizures. One dog showed deterioration due to increased seizure frequency and three dogs were judged as stable. Further improvement was noted after the sessions for 14 dogs, mainly due to improved behavior at home, and according to neurological examinations performed in the clinic (proportion = 0.41 [0.26–0.55], *p* value = 0.85). During the follow-up period, an improvement or normalization of neurological status was observed in all but one seizuring dog (see below).

Thirty-three dogs initially presented with seizures had follow-up characterization of the seizures (frequency and occurrence of cluster seizures/SE). Thirty dogs (30/33, 90.9%) showed reduction of frequency and/or intensity (disappearance of cluster seizures /SE) of seizures (Additional file 1). This corresponded to a significant majority of dogs with reduced seizure (proportion = 0.91 95%CI: 0.82–1.00, *p* value < 0.001). Seizures increased in frequency during RT for one dog, despite absence of peritumoural edema and decrease of tumour size on three consecutive repeat MRI scans over a 10 month’s period. The seizures stopped with aggressive anticonvulsant treatment and the dog was still alive 706 days after the first session without further seizure.

All the dogs that died during the study, showed subacute deterioration of neurologic signs occurring 2 to 42 days before death or euthanasia. For the majority, neurologic signs corresponded to worsening or recurrence of initial clinical signs. For five dogs, new neurologic signs were also observed. Seven dogs underwent follow-up MRI in proximity to clinical deterioration, showing local recurrence and/or suspected metastatic disease (see below).

### Image-based follow-up

At the first recheck, 34 diagnostic control MRI examinations were available for assessment according the RECIST categorization: 21 with initial contrast enhancing tumour (21/34, 61.8%) and 13 without enhancing tumour or presenting non-measurable enhancement (13/34, 38.2%). For dogs with enhancing tumour, response to treatment was Complete Response (CR) in 13/21 (61.9%), Partial Response (PR) in 5/21 (23.8%), and Stable Disease (SD) in 3/21 (14.3%). Progressive disease was not observed. The mean decrease of tumour size was 75% between the diagnosis and the first recheck (decrease of T1-WI+ LD). Taking into account the 13 others non-enhancing tumours, overall 15 cases were classified as CR (15/34, 44.1%) and 19 cases were classified as PR/SD (19/34, 55.9%). All dogs had minimal to marked shrinkage of the mass and a reduction of the mass effect. Additional images show different responses to radiotherapy in an additional file (Additional file 2).

During follow-up, seven dogs that had completed the RT and had normalization of initial clinical signs, presented with clinical signs consistent with brain and/or cervical spinal cord disease, by a median of 229 days after the last session. Local tumour progression and new lesions localized in the brain or in the cervical spinal cord were observed on follow-up MRI in two dogs and five dogs respectively. In those five cases, the initial lesion was stable (1/5), or had shown complete (2/5) or partial (2/5) response. Neurologic signs were consistent with forebrain disease (altered mental status, circling, seizures, 3/5) and/or cervical pain (2/5). These new lesions were considered as metastatic disease. In one dog, neoplastic cells were found on CSF analysis and necropsy revealed metastases of an anaplastic oligodendroglioma in another dog. This dog was the only confirmed histologic diagnosis in our study.

### Survival analysis and prognostic factors

**Fig. 2:**
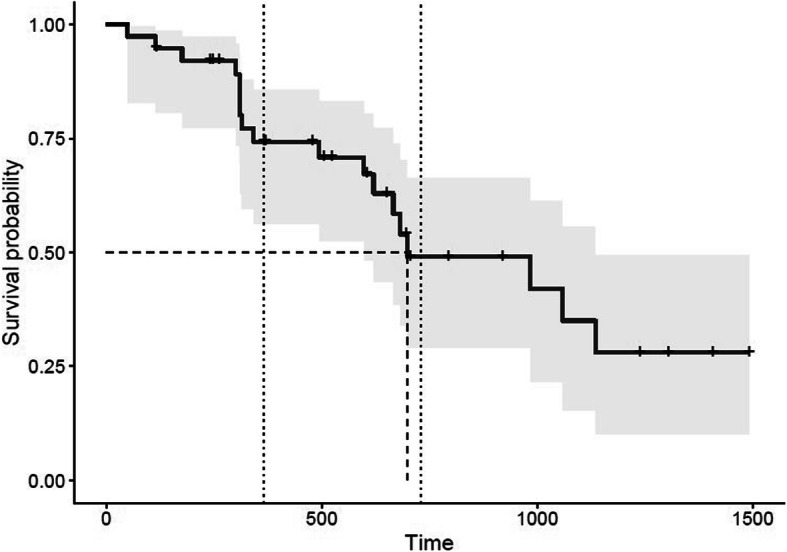
Kaplan-Meier cumulative survival plot for dogs with suspected symptomatic gliomas that had completed the entire RT protocol (per-protocol design, gray zone representing 95% CI). Ticks indicate censored observations. Vertical dotted lines representing 1 and 2 years survival, dashed lines representing median survival time

The median overall survival time (MST) was 698 days (95% CI: 598–1135) (Fig. 2). The estimated survival probability at 1 and 2 years was 74.2 ± 7.4% and 49.0 ± 9.8%, respectively.

#### Univariate analysis

**Fig. 3:**
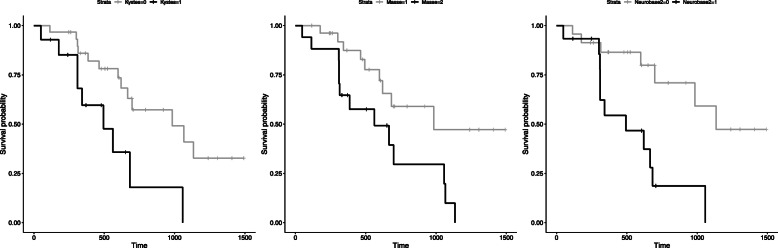
**a**-**c** Kaplan-Meier cumulative survival plot for dogs with suspected gliomas that had completed the entire RT protocol (**a**: according to tumour-mass effect; **b**: according to tumour-cystic pattern; **c**: according to neurological deficits-based scale). Ticks indicate censored observations. Initial neurological signs, MRI-cystic pattern and mass effect have a significant influence on survival (*p < .05)*

Based on univariate analysis, initial neurological signs, MRI-cystic pattern and mass effect showed a significant influence on survival at the level of 5% (Tables 1 and 2). Statistical survival at 1 year and 2 years according to these significant prognostic factors are summarized in Table 3 and KM survival curves are provided in Fig. 3.

**Table 2:**
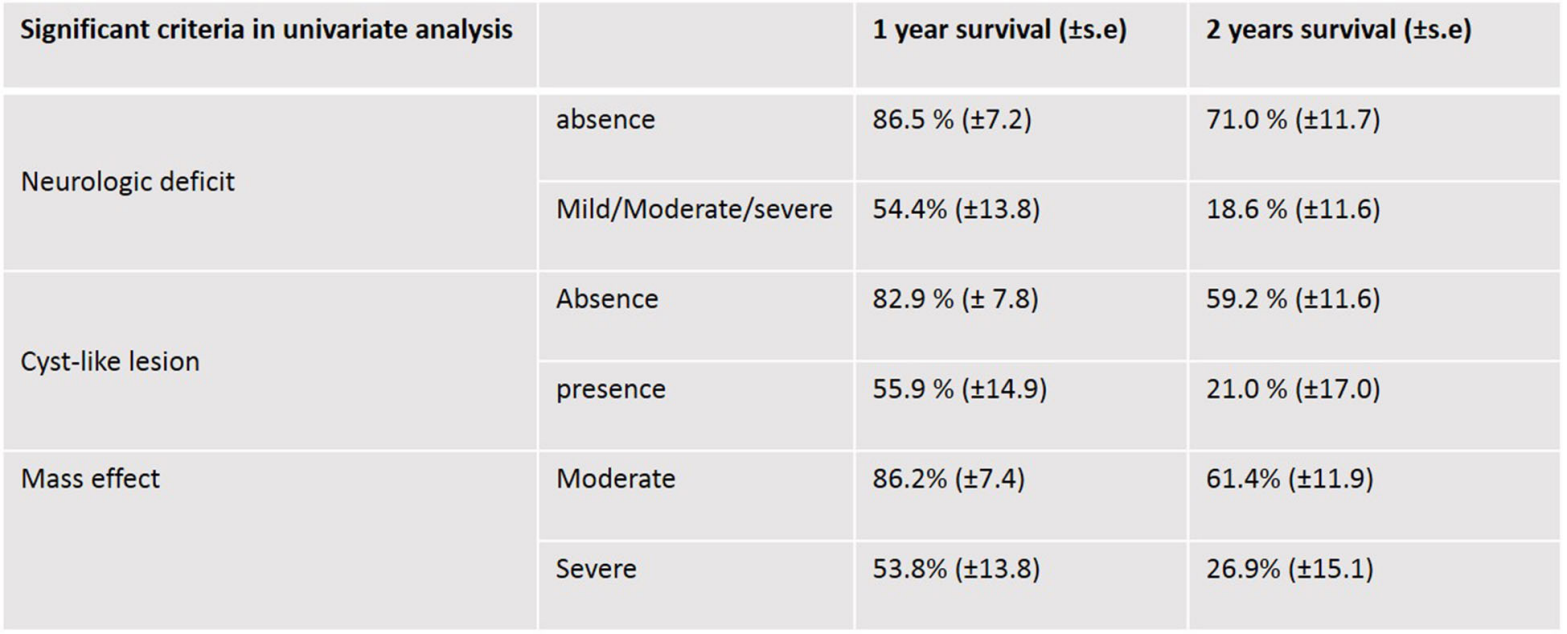
Epidemiological and clinical criteria used for statistical analysis on 38 dogs that had completed the entire RT protocol. Results of univariable statistical analysis on survival (Log-rank tests). Neurologic deficit based scale was adjusted to “presence” or “absence” of clinical signs to allow a significant effect on survival

**Table 3:**
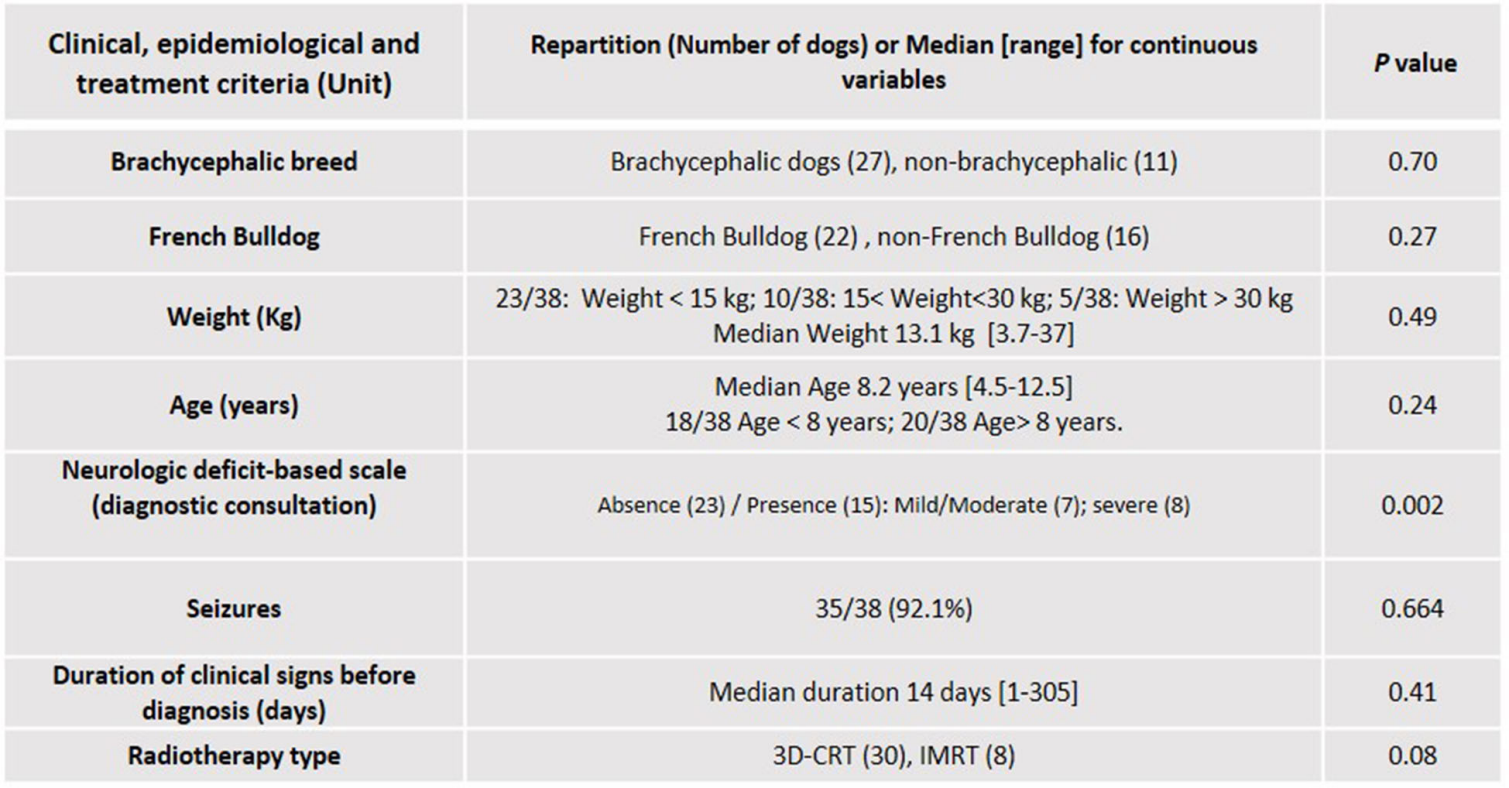
1 and 2 year(s) survival percentages according to statistically significant criteria based on results of univariate analysis (Log-rank tests, *p < .05*; standard error: s.e)

Dogs without neurological deficit observed at diagnostic consultation, or only presenting seizures, had a MST of 1135 days (95%CI: 984-inf), a 2-years probability of survival (S2y) equal to 71.0 ± 11.7% compared to a MST of 494 days (95%CI: 310-inf) and S2y =18.6 ± 11.6% for dogs having initial neurological signs whatever their severity.

Considering significant MRI characteristics, dogs without cystic tumour had a MST of 984 days (95%CI: 666-inf) and a S2y equal to 59.2 ± 11.6% compared to MST of 494 days (95%CI: 309-inf) and a S2y equal to 21.0% ± 17.0% for dogs with cystic tumour. Considering mass effect, dogs with moderate mass effect had a better S2y and a better survival compared with dogs with severe mass effect: 61.4 ± 11.9 versus 26.9 ± 15.1%, and 984 days (95%CI: 620-inf) versus 666 (95%CI: 310-inf) respectively.

#### Multivariate analysis

**Fig. 4:**
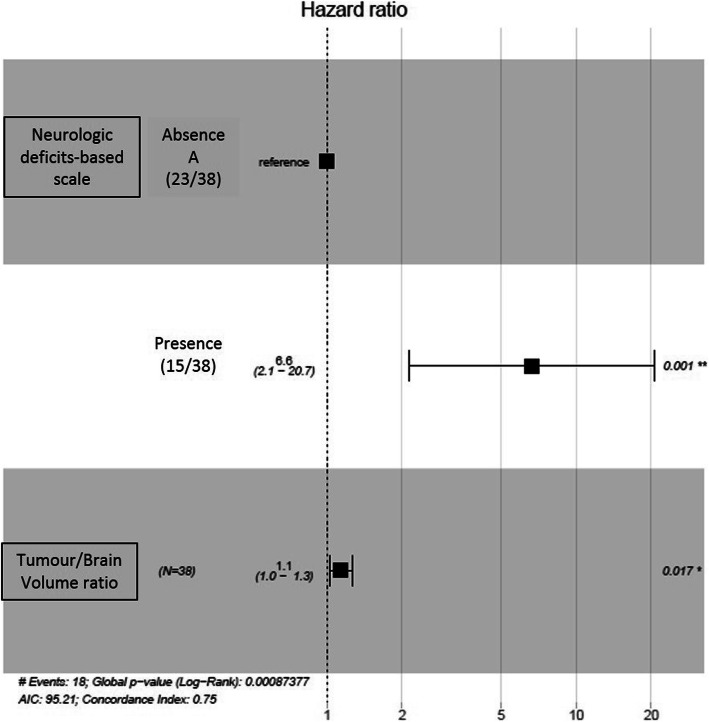
Forest plots of the Hazard ratio and their 95%CI intervals associated with neurologic deficits-based scale and Tumour/Brain Volume ratio

Additional significant variables in the univariate analysis at the level of 20%, which were then included in the multivariate Cox model, were the cortical sulci disappearance, tumour location, edema, T2 hyperintensity homogeneity, Tumour volume, Tumour/Brain Volume ratio and Post-RT/Pre-RT Tumour volume ratio. After step by step selection of variables, neurologic deficits-based scale and Tumour/Brain Volume ratio were the only variables remaining significantly associated with survival (Fig. 4). The regression coefficient estimated for neurologic deficit based scale was 1.89 ± 0.58 (*p* value Wald test = 0.001) corresponding to a Hazard ratio of 6.6, while the regression coefficient estimated for the Tumour/Brain Volume ratio was 0.13 ± 0.05 (*p* value Wald test = 0.02) indicating that a 1% increase in the tumour/Brain Volume ratio is associated with an increase of risk of death by 14%.

### Evaluation of dogs’ quality of life (Qol) based on owner’s opinion

Owner questionnaire was available for 24 owners. Four owners reported a mild weakness of their dog during the first week of radiation, which disappeared at the second week of treatment. Owners’ perception of Qol was assessed with a Likert 10-value scale (1-Could not be worse and 10-could not be better). The median QoL score before diagnosis was 6 (range 3 to 7). After the radiation therapy, the median QoL was 9 (range 5 to 10). Twenty-three responders stated that the QoL of their dog was stable or had improved with the radiation therapy, which corresponded to a significant improvement of QoL score after radiation therapy (one sided Wilcoxon signed rank test z = − 7.96, *p* value< 0.001). Improvement was observed within the first 3 weeks of radiation for the majority of cases (21/24). Among those 21 dogs, 11 (52.3%) were classified as PR (4/11) or SD (7/11) and 19 were receiving corticosteroids treatment for at least 1 week before the first RT session, supposing improvement could already be noticed.

## Discussion

The present study retrospectively described the outcome in 38 dogs treated with a complete uniformly fractionated megavoltage radiation protocol of 15x3Gy. To the author’s knowledge, it is one of the largest studies on external RT as the sole treatment of presumed canine symptomatic intracranial glioma. MST was 698 days for dogs that underwent the entire protocol, with 74.2% of animals surviving more than 1 year and 49.0% more than 2 years. Previous studies evaluating intra-axial tumours, have determined MST following several treatments. In symptomatic protocols (corticosteroids and anticonvulsants) MST doesn’t exceed 3.5 months (35–94 days) [10, 21–23] and surgery doesn’t offer a significant gain in terms of survival [24] compared to symptomatic treatment protocols. Although chemotherapy is often an essential part of the therapy for human patients with intracranial gliomas [25], its benefits remain unclear in veterinary medicine [10, 21, 22]. A recent meta-analysis concerning brain tumour treatment [26], reported a median overall survival time of 226 days for RT-treated intra-axial tumours (range median of 60 to 437 days), based on 127 dogs. Data analysis and interpretation were complicated by the use of different RT methods (orthovoltage, Cobalt radiotherapy, hypo to hyperfractionated). When considering only largest recent studies on megavoltage RT-treated canine intracranial intra-axial tumours, MST variates between 226 and 430 days [9–11, 13]. Disease-specific survival for intracranial intra-axial tumour is available in only one study, and reached 772 days [11]. Then, based on our results, a protocol of 15 fractions of 3 Gy over 5 weeks seems to offer an interesting therapeutic alternative associated with an apparent benefit for life expectancy, compared with symptomatic treatment, without evidence of deleterious life-threatening early/early delayed radiation toxicosis. This protocol was also associated with significant improvement of clinical signs, better control of seizure and lack of evident adverse reactions. This is in accordance with a recent study [27] that demonstrated a significant benefit of external megavoltage radiotherapy on seizure freedom period, compared with symptomatic treatment, on dogs with intracranial tumours.

Because of variability in RT protocols’, study designs’ and populations, comparison with other publications is problematic. Nevertheless, several particularities of our study may have influenced the survival. The majority of actual megavoltage RT protocols applies between 10 to 20 fractions of 2 to 4 Gy, over 3 to 5 weeks, on consecutive or alternate days, making our protocol theoretically comparable in terms of Biologically effective dose (BED, Additional file 3) or toxicity [28]. This protracted protocol with alternate sessions allowed us to space out general anesthesia on fragile dogs with potential high anesthetic risks. Unlike previous reports [10, 11], we applied it homogeneously to a large number of dogs (previous populations studied do not exceed 22 dogs), without taking into account the tumour characteristics (size, imaging grade or location). Moreover, because of a long study period and because all deaths were, potentially, attributable to the tumour/tumour’s treatment, we were able to include animals with longer follow-up and determine a MST comparable to disease-specific survival, that may have influenced the final survival results. Finally, the prolonged use of corticosteroids may also contribute to the results, by reducing perilesional edema and potential secondary radiotoxicity [29]. The impact of a prolonged corticosteroid therapy would need to be proved in a large comparative prospective study on intracranial gliomas. Based on statistical results, the RT delivery method (3D-CRT or IMRT) did not influence survival. This result is in accordance with a recent study evaluating IMRT on primary brain tumours [30] showing comparable survival time and clinical toxicity compared to previously published data on 3D-CRT.

According to the owner’s perception, the QoL of the dogs was significantly improved. Improvement was usually noted during the first 3 weeks of the protocol for most dogs, even for dogs without significant tumour reduction at the first follow-up MRI or those with prior symptomatic treatment. Based on these findings, despite the theoretical prominent delayed effect of radiotherapy on neoplastic cells, an early beneficial effect may exist, and clinical improvement could be expected even during the course of the protocol. This information could be crucial in the owner’s decision to treat, generally in relation with the initial clinical status of their dog. This finding has been already reported [10, 11, 14, 16], and the exact mechanism of this assumed early effect, independent of the tumour-reduction mass effect, is not clearly understood. Actions on microscopic brain infiltration or on peri-tumoural edema or inflammation may be suspected. Serial follow-up MRIs during radiation therapy may reveal early changes, supporting this theory.

In our study, initial clinical signs were statistically related to survival. Two recent publications on canine megavoltage RT, one dedicated to gliomas [10] and one concerning all types of intracranial tumours [13], are in accordance with this observation, even if this finding remains controversial [11, 14, 20] in others studies which consider all types of intracranial tumours. After adjustment for the other variables, the initial neurological sign remains significantly linked with survival. Only dogs presenting with seizures as the initial complaint, will live statistically longer than dogs presenting with other signs, regardless of their severity.

In accordance with the literature, among image-based MRI criteria, the relative tumour-volume [10, 13] was the strongest significant factor associated with survival. In our study, cyst-like lesions and mass effect were significantly associated with poorer outcome in the first-step univariate analysis. MRI cyst-like lesions can mainly be due to necrotic areas or fluid production by the tumour itself [31] and are normally suspected, based on their T1/T2 signals and margins characteristics. We decided not to differentiate necrosis areas with true cystic areas because MRI doesn’t seem to be sensitive enough to allow the specific distinction [31]. Necrosis is a typical feature of histologically high-grade glial tumour [4, 32, 33]. Therefore, MRI cystic-like lesion could reflect the histological grade tumour, and might be associated with survival. However, no statistical association has been demonstrated between MRI cystic-like component and tumour histologic grade [33, 34]. Moreover, to the authors’ knowledge, association between MRI cyst-like lesions and survival has never been studied. Our results may suggest the inclusion of this MRI criterion in future studies.

Unlike previous published data concerning RT-treated meningiomas [16] or all types of intracranial tumours [14], severe mass effect was statistically related to a poor prognosis in our study. This may reflect that, although the tumour size was not statistically significant, the mass effect, resulting from a combination of location, volume, edema, hemorrhage and infiltration characteristics, has to be considered. After step by step adjustment of variables, these variables were not statistically significant in the multivariable analysis and so cannot be considered strong prognostic factors.

Contrast enhancement has been described as a significant criterion associated with high-grade gliomas [4, 34] that can reflect neovascularization, vasodilatation or alteration of the blood-brain-barrier, all features associated with aggressive tumours [35]. In our study, contrast enhancement (presence and characteristics) was not associated with survival, illustrating that other factors might be important to predict histological grade on MRI or, less probably, that histological grade might not be associated with survival in treated dogs. As a consequence, in a context where predictability of glial tumour type or grade is indeed considered moderate with CT, or even with high field MRI [33, 34, 36], only the objectively measured Tumour / Brain Volume ratio may be useful to predict the prognosis without histological analysis.

Several imaging-based brain tumour response criteria have been described and used in veterinary medicine [37], according to human literature and experience. They allow an objective evaluation of therapeutic response and can constitute an important part in the study of clinical management of brain tumours in veterinary patients. Nonetheless, no consensus has been established. In our study, we decided to choose the RECIST criteria implemented by clinical assessment and taking into account the lesion’s T2-WI hyperintensity (excluding suspected peri-tumoural edema). This can allow specific assessment of non-enhancing tumour, which can be observed in canine glial tumours. However, according to statistical analysis, we did not highlight any link between RECIST criteria and survival, not even between T1LD variation and survival, as suspected in a previous study [19]. Therefore, categorization of the response as CR rather than PR or SD might not preclude a long survival time. RECIST criteria may help homogenize therapeutic response assessment in future studies, but its association with survival might not be significant.

Our statistical conclusions are limited by relatively small sample sizes in classification subgroups and small numbers of non-censored dogs as time progresses are associated with larger standard errors. Furthermore, we decided to base our study on a per-protocol design. In order to avoid biases due to study design and selected inclusion criteria, we compared our survival results to an intent-to-treat study design; furthermore, we investigated the Progression Free Survival (PFS). These survival analyses has been performed using Kaplan-Meier curve analysis on a population including the 46 dogs concerned by our protocol during the study period, as mentioned in the results: 38 dogs initially included, two dogs that haven’t completed the entire protocol and six dogs that underwent a second radiation protocol. The results were similar in terms of MST, PFS and survival percentages at 6 months, 1 and 2 years (Additional file 4).

In this study, seven dogs had evidence of progressive disease during the follow-up period, including two dogs with local recurrence and five dogs with meningeal or parenchymal intracranial or spinal cord, multifocal suspected metastasis without evidence of local recurrence. The occurrence of metastatic intracranial glioma is not clearly defined in canine medicine, even more in irradiated animals [10]. In our study, only two dogs had cytological or histological confirmation of the diagnosis of metastatic disease. This finding could be of interest for further studies describing the occurrence of secondary metastatic disease of intracranial gliomas. It might suggest that chemotherapy might be an additional treatment to RT to limit dissemination.

Limitations of this study can be discussed. One is the lack of histopathological analysis to confirm the diagnosis of glioma and their histological grade. However, MRI have been demonstrated to be a sensitive and specific tool to detect neoplastic lesions and diagnose tumour type (extra/intra-axial), particularly gliomas [31, 38], especially when clinical information and epidemiological data are considered [38–40]. However, vascular disease may lead to false diagnosis and cerebrovascular strokes may be misdiagnosed as gliomas [38, 40]. In order to decrease this risk of misdiagnosis, several measures were taken:

- Clinical and epidemiological information for each dog were available for the reviewers.
- The presence of mass effect was an inclusion criterion, which is considered discriminant between ischemic infarct and neoplastic disease [40–42].
- The DWI and signal analysis on ADC Map were incorporated in the MRI characteristics. Although definitive diagnosis cannot be done in Humans or animals based on DWI [43, 44], it may help in the differentiation between gliomas and acute infarction with low ADC and high DWI signal [8, 40]. These MR lesions were therefore excluded.

The retrospective nature of the study did not enable collection and completion of the questionnaires in an anonymous way and at the same time point during the follow-up period.. Furthermore, lack of necropsy did not allow the exact cause of death to be established, nor the detection of tumour recurrence or late radionecrosis, in dogs with neurologic worsening.

## Conclusion

In conclusion, external megavoltage radiation therapy with fractionated protocol (15 × 3 Gy, for 5 weeks), for dogs with primary suspected intracranial glial tumours, provides a long-term tumour control with a reasonably low risk of symptomatic complications and a significant early and persistent improvement in the dogs’ quality of life. In our study, the initial neurologic deficits and the Tumour/Brain Volume ratio are statistically related to survival as in previous studies. Our results also suggest that MRI cyst-like lesions and mass effect may be of prognostic interest.

## Methods

### Study design

Retrospective observational study.

### Dogs and tumour characteristics

The database of the neurology service of the authors’ institution was searched for dogs with presumptive symptomatic intra-axial brain tumours that had been treated with fractionated radiation therapy as a primary treatment modality (between January 2013 and February 2019). The protocol had to be entirely completed. All dogs had full clinical and neurologic examinations performed before the radiation protocol. The presumptive diagnosis of intracranial glioma was made on signalment, history, neurological symptoms and compatible MRI characteristics, based on veterinary neurologist and human radiologist interpretations. MRI features consistent with glial tumour included the identification of a solitary intra-axial lesion exerting a mass effect and characterized by an altered T2 signal [8, 39, 41, 45] with variable contrast enhancement (ranging from none to variably intense, non-uniform or ring-like enhancement) and possible perilesional edema. In order to streamline our data and homogenize final information with prior studies, we decided to consider, inclusion and exclusions criteria, descriptions indices and clinical grading previously described.

The clinical-imaging exclusion criteria were inspired by Dolera [10], and implemented as followed:

- Previous chemotherapy or surgical treatment of the tumour;
- Lesion localization close to a vascular territory of a main cerebral artery or a perforating artery with sharp demarcation [42], strong hyperintensity in the diffusion image and hypointensity on ADC (Apparent Diffusion Coefficient) map [40] suggesting a vascular origin [40, 42, 46];
- Fever/other symptoms or blood abnormalities correlated with inflammation, presence of any predisposing factor to infection, image characteristics of an abscess (T2-Weighted images (T2-WI) hypointense peripheral rim or peripheral concentric “onion skin like” hypointense rim, thick strong peripheral rim contrast enhancement [47] or a granuloma (dural contact, T2-WI hypointensity) [48] and concurrent findings of meningitis [48, 49]; these aspects were considered indicative of inflammatory diseases [38];
- Well defined rounded structure, isolated or in continuity with ventricles and cisterns with thin, smooth non-enhancing walls and content resembling cerebrospinal fluid (CSF) or fat, that did not display contrast enhancement, were considered congenital intracranial cysts (arachnoid or dermoid cysts) [50].

Data from the medical records were reviewed, including breed, age, sex, weight, neurological status and seizure history at the time of presentation, additional treatments and survival time. For evaluation of prognostic factors, the initial clinical signs were classified by a board-certified neurologist, according to a neurological scale based on their severity and the presence or absence of seizures [11, 20, 51] (Table 4).

**Table 4:**
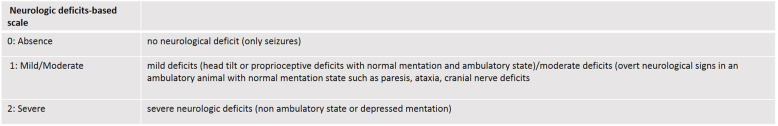
The neurologic deficit-based scale considers neurological abnormalities observed during the neurological examination performed at the diagnostic consultation [11, 20, 51]

All dogs had a complete whole-body CT scan (16-row multidetector helical CT unit GE Healthcare), before and after administration in a cephalic vein of 2 mL/kg body weight of sodium and meglumine ioxotalamate IV, ventilation being manually controlled in all dogs, and scans made at the end of inspiration to evaluate potential co-morbidity, that could modify the therapeutic decision and outcome.

All MRIs were conducted using a 1.5-T MRI scanner (1.5-T GE Healthcare) and performed prior to the beginning of radiation protocol. Imaging sequences were obtained in 3 mm transverse T2-WI, T2 fluid-attenuated inversion recovery (T2-FLAIR-WI), transverse diffusion-weighted echo planar pulse sequence (DWI, b-value 1000 s/mm2), T2*-weighted gradient record echo (T2*-WI) or Susceptibility Weighted images (3D gradient Echo, SWAN sequence), and 0.8 mm 3D-T1-weighted images (T1-WI), before and after intravenous injection of a contrast medium (T1-WI +). For post-contrast images, Gadolinamide (Dotarem; Guerbet, France) 0.05 mmol/mL was administered intravenously at the dose of 0.2 mL/kg.

With regard to tumour characteristics, images were reviewed and 14 descriptive criteria were considered (Table 5). Tumour volume (GTV volume), Tumour/Brain volume ratio and Post-RT/Pre-RT Tumour volume ratio were computed for each dog, when possible, and added to the tumour characteristics reported in the Table 5. Finally, the grade and all the tumour criteria were then evaluated for their influence on prognosis.

**Table 5:**
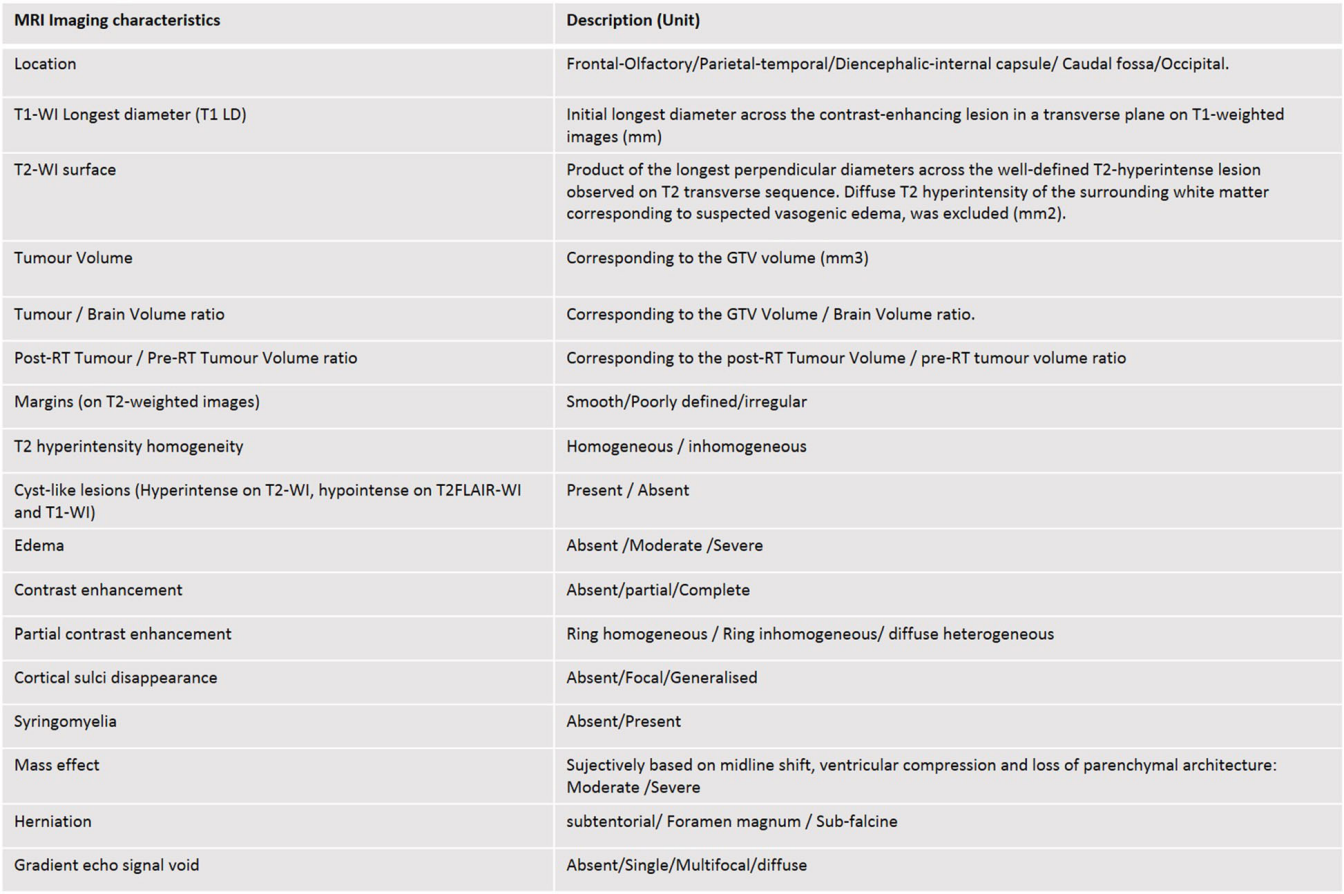
Pre-radiation MRI characteristics used for tumour description and statistical analysis [11, 14, 16, 19]

### Treatment

All dogs were treated with external beam megavoltage RT. Radiation protocol for all dogs consisted of 15 sessions of 3 Gray (Gy), over 5 weeks, on a Monday-Wednesday-Friday schedule to a total dose of 45 Gy. Radiation was delivered with a 6-MV linear accelerator (Clinac iX, Varian, Palo Alto, California) equipped with a 5-mm leaf-width multi-leaf-collimator, using 3D-CRT or IMRT. Additional details on the radiotherapy protocol description are summarized in an additional file (Additional file 3).

All irradiated dogs received approximatively, 0.5 mg/kg/q24, methylprednisolone sodium succinate during the RT protocol until, at least, the first follow-up. Twenty-one dogs were already treated with corticosteroids before the first RT session, 17 dogs had corticosteroid treatment started on first day of protocol. When seizures were reported, anticonvulsant treatment was recorded.

### Clinical and image-based follow up

Clinical and neurological examinations were performed at least once a week during the irradiation period and at the last session of the protocol. If dog presented seizures before RT, seizure characteristics (frequency and occurrence of cluster seizures/ SE) were recorded at the end of RT in order to allow determination of clinical improvement (Additional file 1). After the treatment period, dogs were re-evaluated (clinical and MRI control) for the first time, approximatively 3 months after the last session, then every 2 to 6 months. Changes of neurological signs and corticosteroid therapy were carefully recorded allowing detection of potential early/early delayed radiation toxicity between last session and first recheck. Change of neurological status was then retrospectively evaluated as “stable”, “improved” or “deteriorated” at the last radiation session and at the first post-radiation consultation, based on follow-up informations, gathered from medical records. Specific response evaluation criteria were established to assess regression after irradiation, and observed on the first follow-up MRI. The MRI evaluation was performed inspired by the revised Response Evaluation Criteria in Solid Tumour (RECIST) categorization implemented with clinical status [52]. In case of enhancing lesions, it was based on a one-dimensional tumour measurement of enhancing lesions on transverse T1-WI+ or, in case of non-enhancing lesion, it was based on a two-dimensional tumour measurement on transverse T2-WI [37, 52]. The categorical assignment criteria are reported in an additional file (Additional file 5). For dogs that were not re-evaluated by ourselves, referring veterinarians and owners were interviewed by phone by one of the authors. Death was attributed to the tumour or to the RT, if intracranial neurological signs were reported.

Radiation toxicities were graded according to the Veterinary Radiation Therapy Oncology Group criteria [29, 53] (Additional file 6).

### Evaluation of dogs’ QoL based on owner’s opinion

Information regarding owner’s perception of the benefits of RT was obtained using a standardized questionnaire (Additional file 7). The questionnaire was distributed to the owners after completion of the radiation protocol: at their first re-evaluation, sent by e-mail or fulfilled by telephone with one of the authors, between 2 months and 4 years after the radiation therapy. The goal was to determine owner’s perception of the change in clinical sign and quality of life (QoL) of their dogs before, during, just after RT and during the follow-up period, and evaluate their opinion about their decision to treat.

### Statistical analysis

To test if a majority of dogs showed improvement of their clinical status, the proportion of animal showing improvement for each of the clinical criterion was compared to 0.5 using one-side z-test.

Data were encoded in Excel and analyzed with R (version3.5.3, Vienna, Austria). Animals that didn’t complete the entire RT protocol were excluded from the statistical analysis. All deaths were considered events. Dogs lost to follow-up and dogs alive at the time of reporting were censored. Survival time or time until censoring were defined from the first session of the RT protocol.

In a first step of the analysis, population survival probabilities were calculated using Kaplan–Meier (KM) approach. Log-rank tests were also used to assess in univariate analysis the effect of each cross classified epidemiological, clinical, and imaging variables, as well as radiation therapy type (3D CRT/IMRT) on survival curve. For some factors which survival did not significantly differ, we recodified and gathered classes, keeping biological meaning, in order to reanalyze their statistical significance. Cox proportional hazard model and likelihood ratio test were used in univariate analysis to assess the effect of continuous variables (significant *p* value < .05). In addition, in a second step of the analysis, multivariate analysis was performed using Cox proportional hazard model. All factors with *p* value lower than 0.2 in the first step of the analysis, were included in the saturated Cox model. Variables were then selected by step by step descending procedure using the likelihood ratio test as selection criterion to obtain the reduced Cox model. The proportional hazards assumption was checked using the Schoenfeld residuals for each variable included in Cox models. R Survival package was used for the analysis (*A Package for Survival Analysis in R*. R package version 3.1–12, https://CRAN.R-project.org/package=survival).

Improvement in the QoL was tested by comparing QoL during and after RT using one sided Wilcoxon signed rank test.

## Supplementary information

**Additional file 1.** Clinical improvement at the end of the RT protocol for 30 dogs initially presenting seizures.

**Additional file 2: Figure a.** Right frontal glioma. A, B: Pre-treatment T2-WI (A), T1-WI + (B). C,D: Two months post-RT. T2-WI (C), T1-WI + (D). These images show complete response with disappearance of contrast-enhancement. **Figure b.** Right piriform glioma. A,B: Pre-treatment T2-WI (A), T1-WI+ (B). Absence of contrast enhancement. C,D: 2 months post-RT. T2-WI (C), T1-WI+ (D). These images show PR/SD with decreased T2-WI hyperintensity, according to RECIST criteria for non-enhancing tumour. **Figure c:** Left piriform glioma. A,B: Pre-treatment T2-WI (A), T1-WI+ (B). C,D: Four months post-RT. T2-WI (C), T1-WI + (D). These images show partial response with persistent diffuse contrast enhancement (dotted arrow).

**Additional file 3.** Radiotherapy protocol description.

**Additional file 4.** Intent-to-treat study design: Overall and Progression-Free survival analyses.

**Additional file 5 **RECIST criteria implemented with clinical evaluation [20, 37, 52]: MRI enhancing lesions are defined as enhancing lesions visualized on transverse T1-WI+ (the minimum size was 10 mm). MRI non-enhancing tumours are designed as well delimited T2-hyperintensity (suspected vasogenic edema, corresponding to diffuse T2 hyperintensity of the surrounding white matter, was excluded), visualized on transverse T2-WI. For enhancing tumours, the longest diameter (LD) across the contrast-enhancing lesion on transverse T1-WI+ was measured and reported as the baseline diameter (*T1-WI+ LD*). Non-enhancing tumour’ surface were calculated as the product of the longest perpendicular diameters (*T2 surface*, mm2).

**Additional file 6.** Radiation toxicities, definitions and grades of radiation toxicities used in the study (according to the Veterinary Radiation Therapy Oncology Group) [29, 53].

**Additional file 7.** Owner questionnaire.

## Data Availability

The datasets used and/or analyzed during the current study are available from the corresponding author on reasonable request.

## Abbreviations

3D-CRT: 3-dimensional conformal radiation therapy
ADC: Apparent Diffusion Coefficient
BED: Biologically effective dose
CNS: Central nervous system
CR: Complete response
CSF: Cerebrospinal fluid
CT: Computed tomography
CI: Confidence interval
DWI: Diffusion-weighted images
GTV: Gross volume tumour
Gy: Gray
IMRT: Intensity-modulated radiation therapy
IV: Intravenous
KM: Kaplan-Meier
MRI: Magnetic Resonance Imaging
MST: Median overall survival time
PD: Pogressive disease
PR: Partial response
PTV: Planning target volume
QoL: Quality of life
RECIST: Response Evaluation Criteria in Solid Tumour
RT: Radiotherapy
S2y: 2-years probability of survival
ST: Stable disease
T1LD: T1-WI longest diameter
T2-WI: T2 weighted images
T1-WI: T1 weighted images
T1-WI+: T1 weighted images post-contrast
T2-FLAIR-WI: T2 fluid-attenuated inversion recovery weighted images
T2*-WI: T2* weighted images
