# Supplementary material for: Definitive-intent uniform megavoltage fractioned radiotherapy protocol for presumed canine intracranial gliomas: retrospective analysis of survival and prognostic factors in 38 cases (2013–2019)
Source: BMC Vet Res. 2020 Oct 31;16:412. doi: 10.1186/s12917-020-02614-x (PMC7603708; doi:10.1186/s12917-020-02614-x)
Supplement: Supplementary file 1 — Additional file 1. Clinical improvement at the end of the RT protocol for 30 dogs initially presenting seizures. [file 12917_2020_2614_MOESM1_ESM.docx]

**Clinical improvement at the end of the RT protocol for 30 dogs initially presenting seizures.**

Seizure episode: single seizure or cluster seizures.

Cluster seizures: ≥ 2 seizure episodes within 24 hours.

Status epilepticus (SE): continued seizure activity > 5 minutes or continuous epileptic seizures between, which there is incomplete recovery of consciousness.

Clinical improvement was defined as:

- Prolonged inter-ictal period/Seizure freedom for dogs with duration of clinical signs less than 2 months.
- Decreased monthly seizure frequency (median determined when duration of clinical signs was > 2 months).
- Reduction or absence of SE/cluster seizures for dogs having recurrent SE/Cluster.

| **Type of clinical improvement** | **Number of dogs** |
| --- | --- |
| Prolonged inter-ictal period/Seizure freedom for dogs with duration of clinical signs less than 2 months (inter-ictal period < 5 weeks). | 13 |
| Decreased monthly seizure frequency (median determined when duration of clinical signs was > 2 months). | 12 |
| Reduction or absence of SE/cluster seizures for dogs having recurrent SE/Cluster. | 5 |
