# Supplementary figures and images for: Definitive-intent uniform megavoltage fractioned radiotherapy protocol for presumed canine intracranial gliomas: retrospective analysis of survival and prognostic factors in 38 cases (2013–2019)
Source: BMC Vet Res. 2020 Oct 31;16:412. doi: 10.1186/s12917-020-02614-x (PMC7603708; doi:10.1186/s12917-020-02614-x)

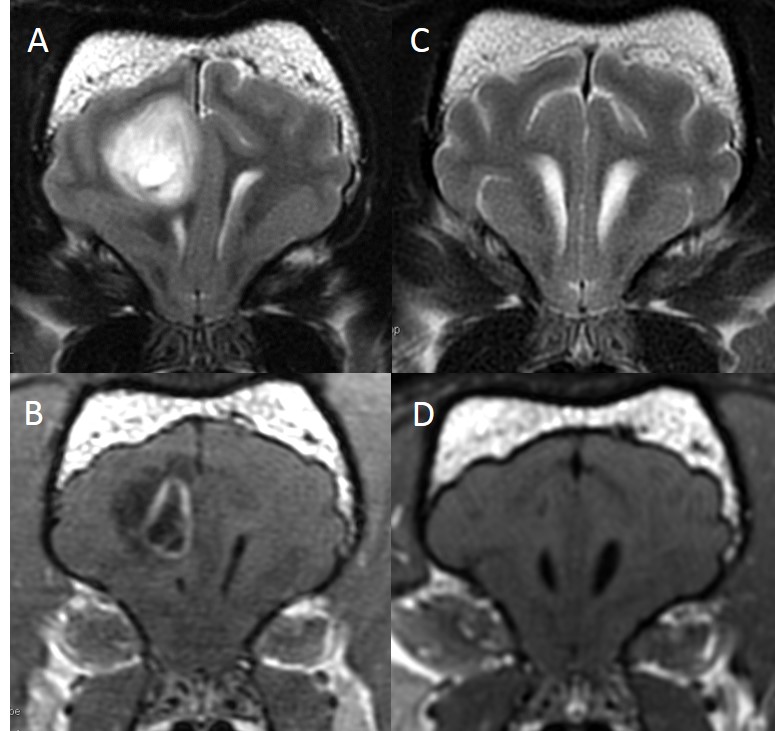

Supplement: Supplementary file 2 — Additional file 2: Figure a. Right frontal glioma. A, B: Pre-treatment T2-WI (A), T1-WI + (B). C,D: Two months post-RT. T2-WI (C), T1-WI + (D). These images show complete response with disappearance of contrast-enhancement. Figure b. Right piriform glioma. A,B: Pre-treatment T2-WI (A), T1-WI+ (B). Absence of contrast enhancement. C,D: 2 months post-RT. T2-WI (C), T1-WI+ (D). These images show PR/SD with decreased T2-WI hyperintensity, according to RECIST criteria for non-enhancing tumour. Figure c: Left piriform glioma. A,B: Pre-treatment T2-WI (A), T1-WI+ (B). C,D: Four months post-RT. T2-WI (C), T1-WI + (D). These images show partial response with persistent diffuse contrast enhancement (dotted arrow). [file 12917_2020_2614_MOESM2_ESM.zip › Additional file 2a.jpg]

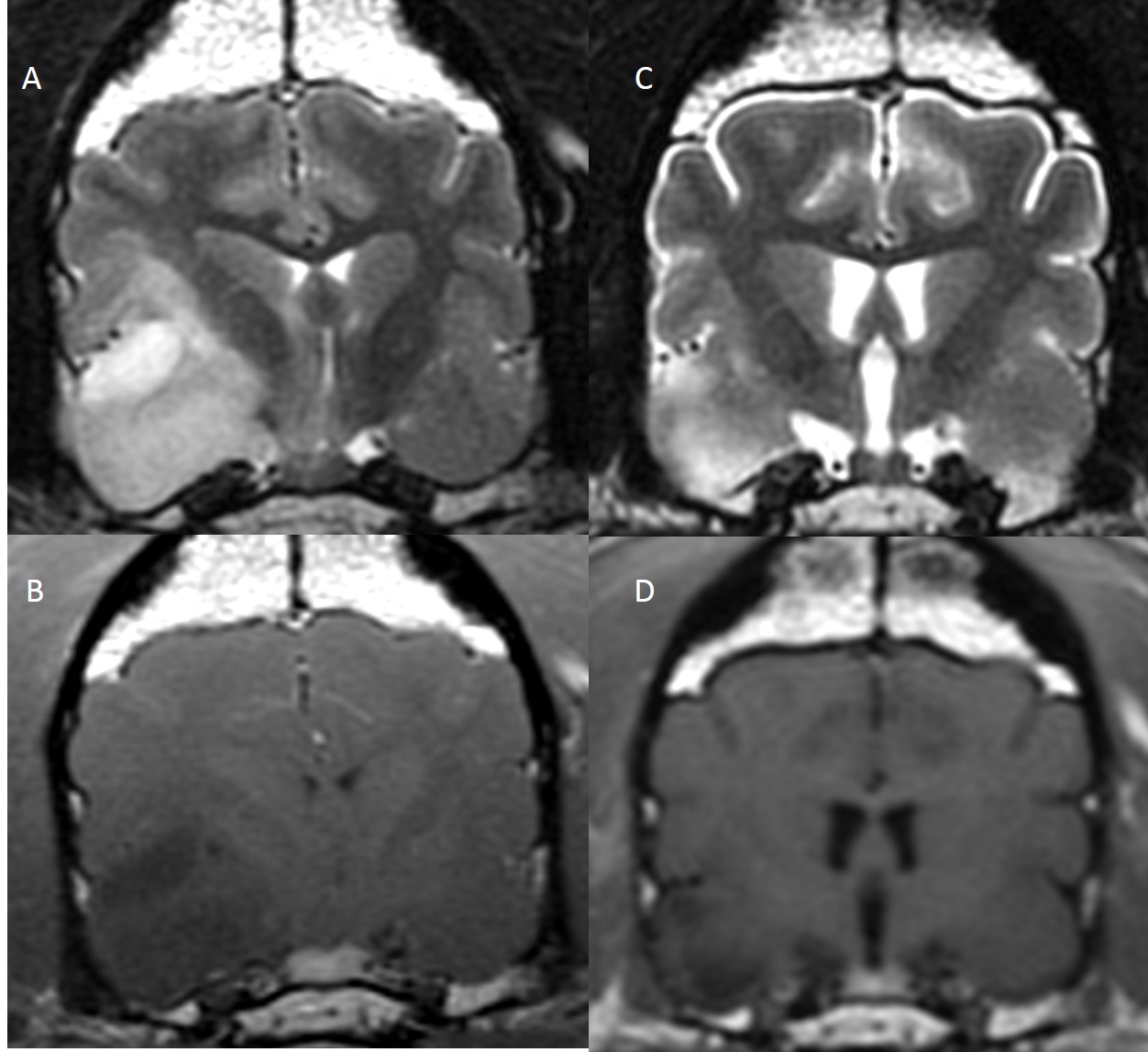

Supplement: Supplementary file 2 — Additional file 2: Figure a. Right frontal glioma. A, B: Pre-treatment T2-WI (A), T1-WI + (B). C,D: Two months post-RT. T2-WI (C), T1-WI + (D). These images show complete response with disappearance of contrast-enhancement. Figure b. Right piriform glioma. A,B: Pre-treatment T2-WI (A), T1-WI+ (B). Absence of contrast enhancement. C,D: 2 months post-RT. T2-WI (C), T1-WI+ (D). These images show PR/SD with decreased T2-WI hyperintensity, according to RECIST criteria for non-enhancing tumour. Figure c: Left piriform glioma. A,B: Pre-treatment T2-WI (A), T1-WI+ (B). C,D: Four months post-RT. T2-WI (C), T1-WI + (D). These images show partial response with persistent diffuse contrast enhancement (dotted arrow). [file 12917_2020_2614_MOESM2_ESM.zip › Additional file 2b.jpg]

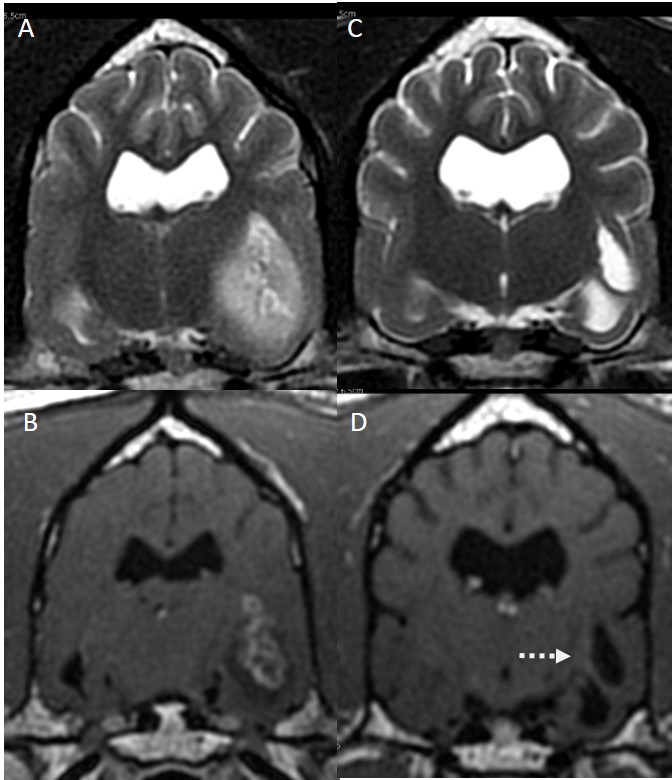

Supplement: Supplementary file 2 — Additional file 2: Figure a. Right frontal glioma. A, B: Pre-treatment T2-WI (A), T1-WI + (B). C,D: Two months post-RT. T2-WI (C), T1-WI + (D). These images show complete response with disappearance of contrast-enhancement. Figure b. Right piriform glioma. A,B: Pre-treatment T2-WI (A), T1-WI+ (B). Absence of contrast enhancement. C,D: 2 months post-RT. T2-WI (C), T1-WI+ (D). These images show PR/SD with decreased T2-WI hyperintensity, according to RECIST criteria for non-enhancing tumour. Figure c: Left piriform glioma. A,B: Pre-treatment T2-WI (A), T1-WI+ (B). C,D: Four months post-RT. T2-WI (C), T1-WI + (D). These images show partial response with persistent diffuse contrast enhancement (dotted arrow). [file 12917_2020_2614_MOESM2_ESM.zip › additional file 2c.jpg]

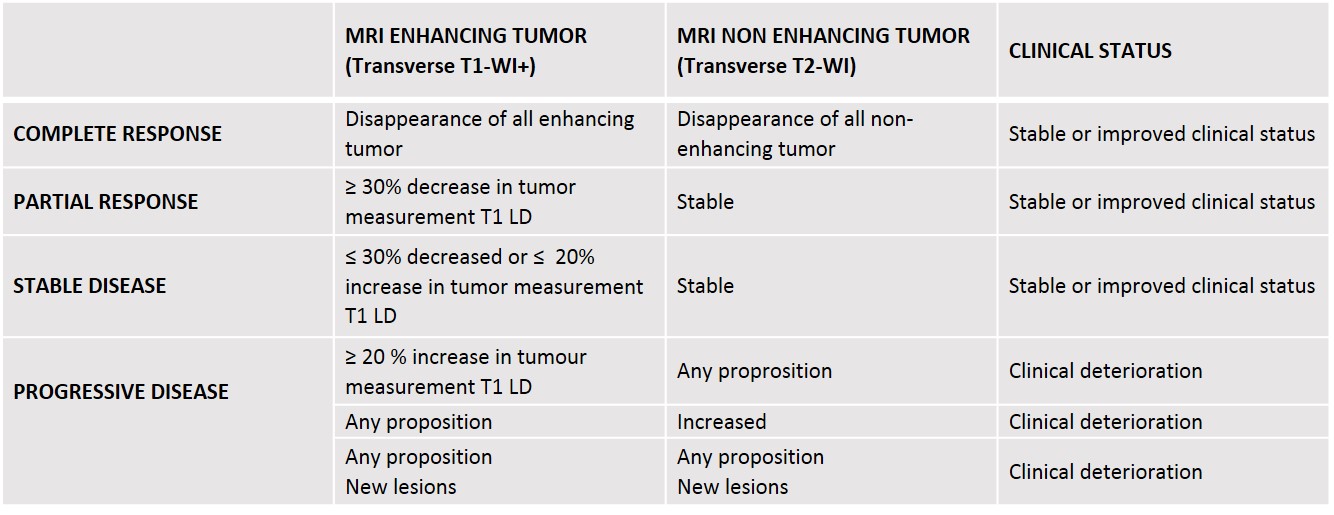

Supplement: Supplementary file 5 — Additional file 5 RECIST criteria implemented with clinical evaluation [20, 37, 52]: MRI enhancing lesions are defined as enhancing lesions visualized on transverse T1-WI+ (the minimum size was 10 mm). MRI non-enhancing tumours are designed as well delimited T2-hyperintensity (suspected vasogenic edema, corresponding to diffuse T2 hyperintensity of the surrounding white matter, was excluded), visualized on transverse T2-WI. For enhancing tumours, the longest diameter (LD) across the contrast-enhancing lesion on transverse T1-WI+ was measured and reported as the baseline diameter (T1-WI+ LD). Non-enhancing tumour’ surface were calculated as the product of the longest perpendicular diameters (T2 surface, mm2). [file 12917_2020_2614_MOESM5_ESM.jpg]

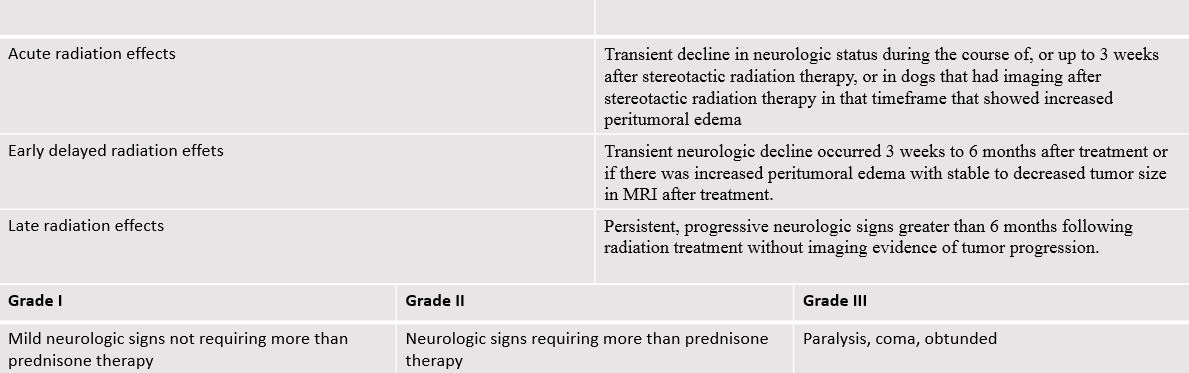

Supplement: Supplementary file 6 — Additional file 6. Radiation toxicities, definitions and grades of radiation toxicities used in the study (according to the Veterinary Radiation Therapy Oncology Group) [29, 53]. [file 12917_2020_2614_MOESM6_ESM.jpg]
