# Supplementary material for: Definitive-intent uniform megavoltage fractioned radiotherapy protocol for presumed canine intracranial gliomas: retrospective analysis of survival and prognostic factors in 38 cases (2013–2019)
Source: BMC Vet Res. 2020 Oct 31;16:412. doi: 10.1186/s12917-020-02614-x (PMC7603708; doi:10.1186/s12917-020-02614-x)
Supplement: Supplementary file 3 — Additional file 3. Radiotherapy protocol description. [file 12917_2020_2614_MOESM3_ESM.docx]

**Radiotherapy protocol description :**

Planning CT scans were performed for each patient in treatment position with an immobilization device adapted to each animal (with a thermoplastic mask and a customized head support, secured to a plastic head-frame with four points of fixation (L-profiles MacroCast^TM^ and ExaFix^TM^ baseplate, Macromedics, The Netherlands), a head rest (Moldcare, Alcare, Japan) and a handmade bite block with thermoplastic granules. Planning-CT and radiation treatments were performed under general anesthesia in sternal recumbency with forelimbs extended caudally and elbow extended. Immobilization and reproducible positioning were accomplished. Target volumes and organs at risk (OAR) were contoured (eyes, optic pathway, normal brain). The gross tumor volume (GTV) was delineated by the same oncologist using contrast enhanced CT images (according the MRI studies) or CT fused with volumic T1-WI+ or volumic T2-WI, in tumors without contrast uptake. Clinical target volume (CTV) accounting for occult or microscopic disease extension of 1 mm was defined. A planning target volume (PTV) was added to include a safety margin accounting for systematic and random uncertainties (PTV margins ranged from 2-4 mm around the CTV in every dimension, in both methods 3D-CRT and IMRT). Accuracy of positioning in every treatments was provided with onboard imaging and systematic orthogonal Megavolt-images (portal imaging) and adjustments were made if necessary. Treatment plans were individually optimized to obtain the best dose distribution for the PTV and the organs at risk as shown by the isodose lines and the dose-volume-histogram, with the intent to include at least 95% of the PTV in the 95-105% isodose, using Eclipse External Beam Planning system (Varian Oncology Systems, Palo Alto California). OAR dose constraints were kept as low as possible according to previous recommandations [54].

IMRT treatment plans were dosimetrically verified before treatment using a QA (Quality Assurance) patient with portal imager (aS500 EPID QA, Portal Dosimetry/ Licence dosimetry review VARIAN/ License ECLIPSE Portal Dose calculation VARIAN Medical System). A gamma index analysis of the isocenter was done for each plan in order to obtain more than 95% of gamma passing rate using a 3% and a 3 mm gamma criterion. The recommendations for specifying dose and volumes as proposed for veterinary medicine were adhered to as proposed in the ICRU reports 50 and 62 for 3DCRT and ICRU report 83 for IMRT plans, and according to Keyerleber et al. [55]. The ICRU reference point was determined at the isocenter for 3D-CRT and at the D median point for IMRT. The originally prescribed dose for all treated cases was 45 Gy at the ICRU reference point, delivered in 15 fractions of 3 Gy.

Radiation plans were derived with a definitive intent for treatment.

Characteristics:

**Biologically effective dose (BED)** according to Bley et al. [44].

**BED= nd [1 + d/ (α/β)] = 15x 3 [1 + 3/10] = 58,5 Gy.**

n: number of fractions = 15

d : dose per fraction = 3 Gy

Value of α/β ratio = 10 for tumours.

- No radiotherapy bolus was used.
- SAD (Source to axis distance): 1000 mm.
- In vivo dosimetry was not performed due to the small size of fields and potential dose loss of 5 to 10% of the dose.
- Physics check: We performed systematic machine check calibration before each day of session.

**Comparison of several recent RT protocols’ BED according to Bley et al. [44]:**

| *Study: First author, Journal, Year of publication.* | *RT protocols:*  *Total dose (Gy)/ Number of fraction* | *BED (Gy)* |
| --- | --- | --- |
| Dolera, Vet Comp Oncol, 2017 [10]. | 42/10  37/7  33/5  35/5 | 59.6  55.5  54.7  59.5 |
| Schwartz, J Vet Med Intern, 2018 [13]. | 50/20  40/10 | 62.5  56 |
| Monforte Monteiro, J Vet Intern Med, 2020 [43]. | 48/12  45/15 | 67.2  58.5 |

**References**

54. Emami B, Lyman J, Brown A, Coia L, Goitein M, Munzenrider JE, Shank B,

Solin LJ, Weeson M. Tolerance of normal tissue to therapeutic irradiation. Int

J Radiat Oncol Biol Phys. 1991;21(1):109–22.

55. Keyerleber MA, McEntee MC, Farrelly J, Podgorsak M. Completeness of

reporting of radiation therapy planning dose, and delivery in veterinary

radiation oncology manuscripts from 2005 to 2010. Vet Radiol Ultrasound.

2012;53(2):221–30.
