# Supplementary material for: Definitive-intent uniform megavoltage fractioned radiotherapy protocol for presumed canine intracranial gliomas: retrospective analysis of survival and prognostic factors in 38 cases (2013–2019)
Source: BMC Vet Res. 2020 Oct 31;16:412. doi: 10.1186/s12917-020-02614-x (PMC7603708; doi:10.1186/s12917-020-02614-x)

**Intent-to-treat study design: Survival analysis**

Progression free survival (PFS) was defined as time from the first irradiation session to the first event of disease progression or death. Patients not known to have progressed or died were censored for PFS. Progression was determined by imaging and clinical criteria:

- An increase in the tumour volume on post-RT follow-up MRI.
- Appearance of any new lesion.
- Clear clinical worsening or death.

|  | **OS percentages [95% CI]** | **PFS percentages [95% CI]** |
| --- | --- | --- |
| **6 months** | 91.1 [78.0-96.6] | 86.9 [73.0-93.9] |
| **1 year** | 76.2 [60.3-86.5] | 69.8 [53.6-81.3] |
| **2 years** | 45.9 [27.9-62.2] | 39.4 [22.4-56.0] |

**Table 1:** Statistical survival percentages according to PFS analysis or OS analysis based on 46 dogs with suspected symptomatic gliomas that had begun the RT protocol.

**Fig.1:** Kaplan-Meier curves for 46 dogs with suspected symptomatic gliomas that had begun the RT protocol, for Progression free survival (PFS) (green curve) and overall survival (OS) (red curve) (Colored zones representing 95% CI).


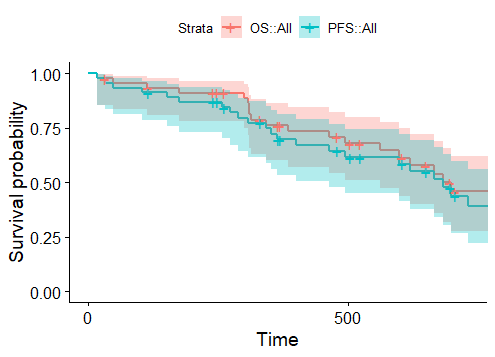

Supplement: Supplementary file 4 — Additional file 4. Intent-to-treat study design: Overall and Progression-Free survival analyses. [file 12917_2020_2614_MOESM4_ESM.docx]
