# Supplementary material for: Definitive-intent uniform megavoltage fractioned radiotherapy protocol for presumed canine intracranial gliomas: retrospective analysis of survival and prognostic factors in 38 cases (2013–2019)
Source: BMC Vet Res. 2020 Oct 31;16:412. doi: 10.1186/s12917-020-02614-x (PMC7603708; doi:10.1186/s12917-020-02614-x)
Supplement: Supplementary file 7 — Additional file 7. Owner questionnaire. [file 12917_2020_2614_MOESM7_ESM.pdf]

Owner Questionnaire:

- What was your pet's quality of life BEFORE the brain tumor was diagnosed:

1-----------2----------3---------4----------5----------6----------7---------8--------9----------10

Quality of life could not be better Quality of life could not be worse

- What is your pet's quality of life AFTER the radiotherapy protocol?

1-----------2----------3---------4----------5----------6----------7---------8--------9----------10

Quality of life could not be better Quality of life could not be worse

- While your pet was receiving radiotherapy, did he/she had any side effects?

a) Yes

b) No

If yes, what were they: ____

- Would you say that your dog had a good tolerance of the radiotherapy:

1-----------2----------3---------4----------5----------6----------7---------8--------9----------10

Not at all Completely

- At the present, how do you feel about treating your pet with radiotherapy?

1-----------2----------3---------4----------5----------6----------7---------8--------9----------10

I regret choosing radiotherapy I am happy that I chose to do radiotherapy

Please expand your answer here ________________________

- Was your animal experiencing seizures as initial neurological signs associated with the brain tumor?

a) Yes

b) No

- If yes, has the seizure frequency been improved after the radiotherapy?

a) Yes b) No

- DURING the radiotherapy protocol, have you noticed:

-- The occurrence or increased behavioural changes like anxiety, aggressiveness, or unusual isolation? a) Yes b) No

If yes, when did you noticed it during the RT protocol: a) First week, b) Second week, c) Third week, d) Fourth week, e) Fifth week.

-- The occurrence of new habits as play games or enthusiastic walks? a) Yes b) No

If yes, when did you noticed it during the radiotherapy protocol: a) First week, b) Second week, c) Third week, d) Fourth week, e) Fifth week.

- AFTER the radiotherapy protocol, have you noticed:

-- The occurrence or increased behavioural changes like anxiety, aggressiveness, or unusual isolation? a) Yes b) No

If yes, when did you noticed it after the radiotherapy protocol: a) Two first weeks, b) 2 to 4 weeks, c) 4 to 6 weeks, d) After 6 weeks.

-- The occurrence of new habits as play games or enthusiastic walks? a) Yes b) No

If yes, when did you noticed it after the radiotherapy protocol: a) Two first weeks, b) 2 to 4 weeks, c) 4 to 6 weeks, d) After 6 weeks.

- AFTER the radiotherapy protocol, would you say your dog regain his youthfulness? a) Yes b) No

- AFTER the radiotherapy protocol, would you say that the initial clinical signs of your dog:

a) Has disappeared

b) Has improved

c) Has been stable

d) Has been increased
